# Risk factors for need of reoperation in bilateral chronic subdural haematomas

**DOI:** 10.1007/s00701-021-04811-5

**Published:** 2021-04-02

**Authors:** Shaian Zolfaghari, Jiri Bartek, Felix Djärf, San-San Wong, Isabelle Strom, Nils Ståhl, Asgeir S. Jakola, Henrietta Nittby Redebrandt

**Affiliations:** 1 Department of Neurosurgery, Institution of Clinical Sciences, Lund University, Lund, Sweden; 2 Department of Neurosurgery, Karolinska University Hospital, Stockholm, Sweden; 3 Department of Neurosurgery, Rigshospitalet, Copenhagen, Denmark; 4 Department of Clinical Neuroscience and Medicine, Karolinska Institutet, Stockholm, Sweden; 5 Department of Neurosurgery, St. Olavs Hospital, Trondheim, Norway; 6 Department of Neurosurgery, Sahlgrenska University Hospital, Gothenburg, Sweden; 7 Department of Clinical Neuroscience, Institute of Neuroscience and Physiology, University of Gothenburg, Sahlgrenska Academy, Gothenburg, Sweden

**Keywords:** Chronic subdural haematoma, Bilateral, Outcome, Recurrence

## Abstract

**Background:**

Chronic subdural haematoma (CSDH) is one of the most common neurosurgical diseases. A subtype of CSDH is bilateral chronic subdural haematoma (bCSDH) which represents 20–25% of patients with CSDH and has a higher recurrence rate. There is no clear consensus on how bCSDH should be treated regarding upfront unilateral- or bilateral evacuation of both haematomas. The purpose of this study was to identify risk factors associated with reoperation of bCSDH.

**Methods:**

A total of 326 patients with radiological evidence of bCSDH were included in this retrospective cohort study where 133 (40.8%) patients underwent primary bilateral evacuation and 193 (59.2%) primary unilateral evacuation. The two centres operated using different surgical approaches. Analyses were performed to identify risk factors associated with reoperation of bCSDH. Reoperation rate was defined as reoperation of CSDH on either side of the hemisphere within 3 months after primary evacuation.

**Results:**

The cohort had a total reoperation rate of 26.4%. Patients which underwent unilateral evacuation had a reoperation rate of 32.1%, and the bilateral group had a reoperation rate of 18.0% (*p*=0.005). Multivariable logistic regression identified unilateral evacuation (OR 1.91, *p*=0.022) and complications according to Ibanez (OR 2.20, *p*=0.032) to be associated with the need of reoperation of bCSDH. One-burr hole craniostomy with active subgaleal drain was primarily performed in bilateral approach (69.4%) whereas patients operated with minicraniotomy with passive subdural drain were primarily operated by unilateral evacuation of the larger symptomatic side (92.8%).

**Conclusions:**

Unilateral evacuation of bCSDH was associated with a higher risk for reoperation than upfront bilateral evacuations in this study. There is a need to further discuss the criteria for uni- or bilateral evacuation since patients are treated differently at different centres.

## Introduction

Chronic subdural haematoma (CSDH) is a common neurosurgical condition with an incidence of 8 to 14 per 100,000 person-years [13]. Common risk factors for CSDH are falls, alcohol abuse, anticoagulant therapy, advanced age and brain atrophy [13]. Furthermore, it has also been shown that antiplatelet therapy alone and in combination with anticoagulant therapy also increases the risk for CSDH [11, 12]. The incidence of CSDH is expected to significantly rise in the coming decades; thus, research concerning the clinical management of CSDHs is of importance [1, 4]. The recurrence rate of CSDH is commonly accepted to be around 10–20% with wide variety between results reported from different centres [5, 7]. The perioperative management of CSDHs varies widely between different centres with the major differences in surgical techniques and types of postoperative drainage [22].

A subtype of CSDHs is bilateral chronic subdural haematoma (bCSDH). There is no consensus on how bCSDHs should be managed due to lack of evidence. Depending on factors such as haematoma size and lateralisation of symptoms, certain centres or neurosurgeons will opt to primarily evacuate unilaterally, while other centres will opt to primarily evacuate bilaterally. Thus, not only resulting in the question of whether the patient should be evacuated upfront with unilateral or bilateral approach, but also if the condition should be treated with one procedure or if two procedures will be required in the case of initial unilateral evacuation.

There has recently been a surge in research concerning bCSDH in order to find evidence for standardised treatment guidelines [3, 16, 19, 24]. It has previously been highlighted that bCSDHs themselves are risk factors for recurrence [3, 5, 10, 14]. One recent study found that upfront bilateral evacuation significantly lowered the risk for reoperation and complications [3]. However, there has also been recent data presented indicating that unilateral evacuation of bCSDH can be sufficient as a management strategy [16]. Overall, there is currently conflicting evidence in literature as to the surgical management of bCSDH.

The aim was to determine risk factors for the need of reoperation of bCSDH with data from two neurosurgical departments in Sweden, with emphasis on the effect of unilateral versus bilateral evacuation.

## Methods and materials

### Study cohort

The following retrospective study cohort included patients with radiological evidence of bCSDHs that underwent surgical evacuation from two anonymised university hospitals in Sweden represented as hospital A and hospital B. The patients at hospital A were operated between 2012 and 2016, and the patients from hospital B were operated between 2006 and 2014. The same surgical protocols were routinely used in the respective centres during the study time periods without any alterations during the study time periods. All included patients were over the age of 18. Patients with unilateral CSDH, cerebral shunts, simultaneous intracranial haemorrhages and patients with permanent residency outside of Sweden were excluded from the cohort. A total of 326 patients were included in this retrospective study cohort.

### Surgical techniques

Patients were operated and managed with the following surgical techniques denoted as method 1 and 2. Note that a minority of the patients included were operated with alternative techniques. Method 1 (i) was the standard surgical technique for evacuation of CSDH at hospital A. Method 2 (ii) was the standard surgical technique for evacuation of CSDH at hospital B.

i. Two or three burr-holes combined into a minicraniotomy: the haematoma was evacuated with irrigation after which a passive drain was placed in the subdural space for 24 h.
ii. Singular burr-hole: the haematoma was evacuated with irrigation after which an active (suction) drain was placed in the subgaleal space for 24 h [22].

### Variables

To assess baseline characteristics for the study cohort, the following variables were included: age, hospital (A or B), gender and Charlson Comorbidity Index (CCI) variables (index which predicts mortality based on weighted comorbidities) [17]. Furthermore, preoperative neurological deficits, presenting Glasgow Coma Scale (GCS) score, antithrombotic medication and anticoagulant medication (vitamin K antagonist and novel oral anticoagulants (NOAC)) were registered. Patients were examined as to thrombocytes, INR, APTT and, in some cases, ROTEM and/or Multiplate. Patients with abnormal test values indicative of coagulopathy were optimised prior to surgery. All patients were examined with a preoperative computed tomography (CT) of the head.

The following surgery-related variables were gathered: midline shift, haematoma diameter on the axial plane on both sides, haematoma density on head-CT (hyperdense, isodense, hypodense and mixed densities), primary surgical evacuated side (unilaterally or bilaterally), surgical procedure performed (craniotomy, 1-burr hole craniostomy (1-BHC), minicraniotomy or other surgical method) and drainage system (active subgaleal drain or passive subdural drain).

Reoperation was defined as reoperation of CSDH on either side of the hemisphere within 3 months after primary evacuation, to account for both haematoma recurrence on the previously operated side and the possible need to operate the contralateral side to the one previously operated. Complications were registered according to Landriel Ibañez (Classification system for complications after neurosurgical procedures) [15]. Reoperation was not registered as a complication in our study.

### Statistical analysis

All statistical analyses were performed using IBM SPSS Statistics for Windows, version 25.0, Armonk, New York, IBM Corporation. Univariate (independent samples *t* test), bivariable (chi-squared and Fisher’s exact test) and multivariable analyses were performed to determine if any of the studied variables were associated with an increased reoperation rate when comparing bilateral and unilateral surgical approach. Odds ratio (OR) for Fisher’s exact test was calculated within a 95% confidence interval. Multivariable logistical regression model with forward selection was used in this study. In the final multivariable models, we only included variables that yielded a *p* value of < .25 in univariate or bivariable analyses. The included independent variables were tested for multicollinearity by analysing variance inflation factor (VIF); a conservative cut-off for multicollinearity was set at a VIF value of over 2.5. Alpha level of significance was defined as *p* value < .05 in all other analyses.

## Results

### Baseline demographics

In total, 326 patients met the inclusion criteria for study participation and were included. All 326 patients had radiological evidence of bCSDH. Out of the 326 included patients, 133 (40.8%) underwent primary bilateral evacuation and 193 (59.2%) bCSDH patients underwent primary unilateral evacuation (Tables 1 and 2). Patients undergoing unilateral evacuations had a mean midline shift of 5.3 mm while bilateral evacuations had a mean midline shift of 3.5 mm (*p*=0.001, independent samples *t* test).

**Table 1:** Baseline characteristics of patients with bCSDH. Variables are stratified for unilateral and bilateral evacuation

Variable, No. (%)	Unilateral evacuation *N*=193 No. (%)	Bilateral evacuation, *N*=133 No. (%)
Hospital		
Hospital A, *n*=153 (46.9)	140 (72.5)	13 (9.8)
Hospital B, *n*=173 (53.1)	53 (27.5)	120 (90.2)
Male, *n*=247 (75.8)	136 (70.5)	111 (83.5)
Mean age (years) ± SD	75.2 ± 10.9	74.3 ± 10.1
Charlson Comorbidity Index		
Score over 1, *n*=123 (37.7)	70 (36.3)	53 (39.8)
Antithrombotic treatment, *n*=68 (20.9)	38 (19.7)	30 (22.6)
Anticoagulation treatment, *n*=68 (20.9)	44 (22.8)	24 (18.0)
Symptoms at presentation (note that patients often had more than one presenting symptom)		
Gait disturbance and falls	127 (65.8)	69 (51.8)
Limb weakness	46 (23.8)	41 (30.8)
Headache	34 (17.6)	50 (37.5)
Speech impairment	38 (19.7)	18 (13.5)
Mental deterioration	23 (11.9)	30 (22.6)
Acute confusion	15 (7.8)	16 (12.0)
Vomiting	11 (5.7)	8 (6.0)
Seizure	7 (3.6)	4 (3.0)
Incontinence	5 (2.6)	3 (2.3)
Visual disturbance	5 (2.6)	2 (1.5)

*SD* standard deviation

**Table 2:** Surgical and outcome data of bCSDH patients. The data in the following table is stratified for unilateral and bilateral evacuation

Factor No. (%)	Unilateral evacuation *N*=193 No. (%)	Bilateral evacuation *N*=133 No. (%)
Mean midline shift (mm) ± SD^a^	5.3 ± 4.0	3.5 ± 2.9
Mean haematoma diameter (mm) ± SD		
Right side	15.7 ± 7.2	15.9 ± 5.4
Left side	16.7 ± 6.9	15.5 ± 5.4
Median GCS score	15	15
CT hematoma density^b^		
Hyperdense	26 (19.7)	21 (16.9)
Isodense	65 (49.2)	60 (48.4)
Hypodense	36 (27.3)	37 (29.8)
Mixed	5 (3.8)	6 (4.9)
Surgical procedure		
1-Burr hole craniostomy with active subgaleal drain, *n*=173 (53.1)	53 (27.5)	120 (90.2)
Minicraniotomy with passive subdural drain, *n*=138 (42.3)	128 (66.3)	10 (7.5)
Other surgical method^c^, *n*=15 (4.6)	12 (6.2)	3 (2.3)
Mean time with drainage system (days)^a^	1.07 ± 0.31	1.02 ± 0.19
Reoperated within 3 months after primary evacuation, *n*=86 (26.4)	62 (32.1)	24 (18.0)
Complications according to Ibañez, *n*=54 (16.6)	43 (22.3)	11 (8.3)
Grade Ia: Complication requiring no drug treatment	10 (5.2)	3 (2.2)
Grade Ib: Complication requiring drug treatment	12 (6.2)	1 (0.8)
Grade IIa: Complication requiring intervention without general anaesthesia	13 (6.7)	1 (0.8)
Grade IIb: Complication requiring intervention with general anaesthesia	4 (2.1)	2 (1.5)
Grade IIIa: Complication involving single organ failure and ICU care.	1 (0.5)	1 (0.8)
Grade IV: Complication resulting in death	3 (1.6)	3 (2.2)

^a^*SD* standard deviation

^b^Data could only be retrieved for 256 patients out of 326.

^c^Other surgical methods consisted of craniotomy and twist-drill craniostomy

### Univariate analysis of complications associated with unilateral or bilateral evacuation

Complications according to Ibañez were dichotomised into a simplified categorical variable containing patients which had experienced a complication and those that did not. Complications graded as Ia, thus those that did not require any treatment, were not included in this variable as they were deemed trivial. Complications were found to be significantly associated with unilateral approach. (*p*=0.001, Mann-Whitney).

### Univariate analysis of factors associated with reoperation of bCSDH.

Univariate analysis was performed on potential factors associated with reoperation of bCSDH which are summarised in Table 3.

**Table 3:** Univariable analysis of potential factors associated with the retreatment of bCSDH (*n*=326). Cut-off for significance was set to *p* < .25, variables which met the significance level were candidates for inclusion in the final multivariable model

Variable	No reoperation	Reoperation	*p* value	CI 95%
Age in years, mean ± SD	74.75 ± 10.76	75.14 ± 10.13	0.770	−3.011, 2.232
Male	177 (71.7)	70 (28.3)	0.187	0.842, 2.879
CCI score over 1, *n* (%)	97 (78.9)	26 (21.1)	0.119	0.377, 1.083
Antithrombotic use, *n* (%)	53 (77.9)	15 (22.1)	0.440	0.395, 1.407
Anticoagulation use, *n* (%)	51 (75.0)	17 (25.0)	0.877	0.494, 1.688
Midline shift (mm), mean ± SD	4.61 ± 3.63	4.35 ± 3.90	0.569	−0.656, 1.193
Surgically evacuated side—unilateral, *n* (%)	131 (67.9)	62 (32.1)	0.005	1.174, 2.699
Surgical procedure—Minicraniotomy with passive subdural drain^a^, *n* (%)	87 (63.0)	51 (37.0)	0.001	1.853, 5.422
Time with drainage system (days), mean ± SD	1.03 ± 0.21	1.09 ± 0.40	0.127	−0.121, 0.015
Complications (not including repeated evacuation of CSDH on either side), *n* (%)	22 (53.7)	19 (46.3)	0.004	1.435, 5.503

*SD* standard deviation

^a^15 cases were excluded due to surgical evacuation with craniotomy and twist-drill craniostomy

Surgical procedure was found to be statistically significant when comparing the two surgical techniques with reoperation within 3 months (*p*<0.001, Fisher’s exact test, OR CI 95% 1.853–5.422). Complications according to Ibañez were dichotomised into a simplified categorical variable containing patients which had experienced a complication and those that did not. Complications graded as Ibañez Ia were not included in this variable as they were deemed trivial. Univariate analysis of complications with reoperation found a significantly higher rate of complications in patients that were reoperated within 3 months (*p*=0.004, Fisher’s exact test).

In our study population, we found a reoperation rate of 26.4% within 3 months of primary surgical evacuation. Patients which underwent primary bilateral evacuation of the bCSDH had a reoperation rate of 18.0% while patients which underwent primary unilateral evacuation had a reoperation rate of 32.1% (*p*=0.005, Fisher’s exact test, OR CI 95% 1.174–2.699).

### Multivariable analysis of factors associated with reoperation rate

Multivariable logistic regression analysis was performed on variables which had a *p* value of < .25 in the univariable analyses. Thus gender, CCI score, surgically evacuated side, surgical procedure, time with drainage and complications were included. Forward selection was employed and yielded only surgical procedure as significant. In this model, patients were 3.17 times more likely to be re-operated when operated with minicraniotomy with passive subdural drain (*p*=0.001, CI 95% 1.838–5.451), and they were for the absolute majority of times operated unilaterally. Correlative measures were calculated, and it was found that the surgical procedure was significantly correlated with the surgical approach, i.e. uni- or bilateral evacuation (Spearman’s *ρ*=−0.626). Collinearity diagnostics was performed on all the independent variables in the multivariable analysis. The analysis did not indicate presence of multicollinearity (VIF< 1.7 for all independent variables).

Due to the significant correlation between surgical procedure and surgical approach but also skewed data in variable surgical procedure (only *n*=10 patients underwent bilateral evacuation with minicraniotomy), a second model using forward selection was created where the variable surgical procedure was excluded in the analysis. From this model, complications and surgical approach (uni- or bilateral evacuation) were found to be significant. Patients which underwent unilateral evacuation were 1.91 times more likely to be re-operated (*p*=0.022, CI 95% 1.097–3.307). Patients which did have complications were 2.20 times more likely to be re-operated (*p*=0.032, 95% CI 1.070–4.521). Correlation between complications and surgical approach was of no significance (Spearman’s *ρ*=−0.164), and collinearity diagnostics did not indicate presence of multicollinearity in this model (VIF < 1.1 for all independent variables)

## Discussion

In this retrospective cohort study, we observed a significantly higher risk of reoperation of bCSDH when treated with unilateral compared to bilateral haematoma evacuation. Patients with complications graded higher than Ibañez Ia had a higher risk of reoperation. Further, we found that complications graded higher than Ibañez Ia were associated with unilateral evacuation, and the group we previously noted had an increased reoperation rate. In addition, minicraniotomy and subdural drainage had unfavourable results compared to 1-BHC and subgaleal drainage, but due to positive correlation with uni- or bilateral treatment and lack of patients operated bilaterally with minicraniotomy, further elaboration was not possible. Patients who underwent primary unilateral evacuation had a significantly larger midline shift, but the size of the midline shift was not a significant factor contributing to the risk of reoperation. From a clinical point of view, it seems reasonable to perform a unilateral evacuation if one of the chronic subdural haematomas is causing a significant midline shift, but we do not have any data here to support how the individual surgeon was motivating the choice to perform uni- or bilateral evacuation.

Our results are most likely not indicative of a problem with minicraniotomy as surgical technique, but rather pointing to the effect of the choice of primary unilateral and bilateral approach. There’s also evidence pointing to the minicraniotomy having a place in the treatment of recurrent more organised CSDHs [2, 13, 18, 25]. Identical recurrence rates have been described previously in other studies [3, 19]. It is also important to note that intuitively, the reoperation rate of bCSDH will be higher compared to unilateral CSDH since there are two CSDHs. The fact that patients with unilateral treatment more often experience complications may be due to the higher reoperation rate in this group of patients, resulting in more time spent in hospitals. This in turn could be due to more re-operations, each one increasing the risk of getting a complication.

The clinical question concerning whether bCSDH should be unilaterally or bilaterally evacuated has been raised in several studies with varying conclusions. A retrospective Danish study including 291 patients published in 2016 displayed results pointing to a benefit in bilateral evacuation with a number needed to treat of approximately 6.8 [3]. Our study confirms and is in line with the results observed in the Danish study with unilateral evacuation of bCSDH significantly increasing the risk of reoperation. Our study is to our knowledge the largest retrospective study which has investigated bCSDH concerning the reoperation rate with respect to the choice of surgical approach [3, 16, 19].

Recently, a retrospective study including 128 patients points to satisfactory outcomes with primary unilateral evacuation of bCSDH given that certain prerequisites were met such as a smaller asymptomatic contralateral side. The authors also identified thickness of the untreated contralateral haematoma and the degree of reversal of midline shift on postoperative CT to be factors predicting the need of surgical evacuation of the opposite (non-operated) side [14]. Important to note however is that the assessment of midline shift reversal only can be done postoperatively; thus, there are difficulties in employing the strategy in a setting where we wish to identify patients in need of upfront bilateral evacuation in order to avoid multiple unilateral evacuations. A British study was also able to show a symptomatic recurrence rate of 9% in treatment of bCSDH with approximately 78% undergoing bilateral evacuation [6]. The symptomatic recurrence rate for bCSDHs was identical to the symptomatic recurrence rate for unilateral CSDHs in the same study (8.2%). The recurrence rates are noticeably much lower than ours which warrants attention. The study did not report data on haematoma volumes for the included patients, which makes more specific comparisons difficult. The study did however only include symptomatic recurrences and only within 60 days of primary evacuation. In our study, we set the time frame to 90 days and did not specify that the indication for re-operations strictly needed to be clinical symptoms, but rather every patient who needed a re-operation, irrespective of the clinical motivation, was included.

Our study population was properly adjusted in both the unilateral and bilateral groups in regard to the haematoma volumes. Taking this into consideration, there seems to be a critical volume of when a haematoma poses a significant risk of expanding and necessitating surgical evacuation. Guidelines will often point to a radiological criteria diameter of 1.5 cm to indicate the need of surgical evacuation. However, we would suggest consideration of bilateral evacuation even when the contralateral side to the symptomatic lesion is below 1.5 cm with the data in hand from this study.

Some studies have tried to quantify and isolate the volume of the contralateral side to predict the need of bilateral evacuation. Two studies found that contralateral haematomas with a volume consisting of more than 40 cm^3^ and 37.84 cm^3^ respectively warranted consideration for bilateral evacuation [19, 21]. As has been previously postulated in several studies, there seems to be a relationship between the two haematomas in bCSDH. It has been intuitively suggested that when performing unilateral evacuation in bCSDH, the decrease in ICP and expansion of the displaced brain could lead to further progress of the smaller CSDH on the non-operated side which would eventually result in the development of symptomatology/neurological deficits. Furthermore, a study was published in 2010 where they observed a significantly slower re-expansion rate of the brain in bCSDH postoperatively compared to unilateral CSDH [14]. It is postulated that the slower brain re-expansion rate allows for shifting of the brain which in turn may result in an increased risk of bridging vein tears; this in turn could explain the higher reoperation rate in bCSDH. This leads to the question of whether it is the reintroduction of the increased subdural space or the active inflammatory cascade reaction that eventually leads to the advancement of the contralateral haematoma [8, 9, 23]. The most likely explanation is that it is a combination of both ‘disease states’, with the contribution of inflammatory reaction causing the continual recruitment and rebleeding by neocapillaries and on the other hand the neurophysiological properties of the brain such as the effect of persistent increased subdural space.

There are also other risk-benefit and socio-economic aspects to consider when evaluating the pro- and cons of uni- vs. bilateral surgery in bCSDH. A potential disadvantage is the increased risk of postoperative pneumocephalus after bilateral reoperation [20]. With bilateral evacuation, there are however several advantages, such as lower cost of upfront bilateral evacuation instead of two separate unilateral procedures as well as less patient-related discomfort.

To our knowledge, this is the largest retrospective study which has analysed risk factors concerning bCSDH with specific emphasis on reoperation. We need clearer indications for bilateral evacuation, such as a minimum established volume on the contralateral side and/or minimum established diameter of the contralateral haematoma. The study is inherently limited by the retrospective design. There are also limitations pertaining to the data quality and dyssynchronous study time periods.

## Conclusion

In this retrospective study, unilateral surgical evacuation of bCSDH seemed to be associated with more reoperations than upfront bilateral evacuation. In addition, there was a higher risk of complications in patients which underwent unilateral evacuation compared to bilateral evacuation. Prospective studies in the form of randomised controlled trials could be of value to establish concrete indication(s) for bilateral evacuation.

## Abbreviations

CSDH: Chronic subdural haematoma
bCSDH: Bilateral chronic subdural haematoma
1-BHC: 1-Burr hole craniostomy
